# Determinants of replication protein A subunit interactions revealed using a phosphomimetic peptide

**DOI:** 10.1074/jbc.RA120.016457

**Published:** 2021-01-13

**Authors:** Sungjin Lee, Jeongbeen Heo, Chin-Ju Park

**Affiliations:** Department of Chemistry, Gwangju Institute of Science and Technology, Gwangju, Republic of Korea

**Keywords:** replication protein A, phosphorylation, fluorescence polarization, protein-protein interaction, DNA-binding protein, nuclear magnetic resonance (NMR), DNA damage response

## Abstract

Replication protein A (RPA) is a eukaryotic ssDNA-binding protein and contains three subunits: RPA70, RPA32, and RPA14. Phosphorylation of the N-terminal region of the RPA32 subunit plays an essential role in DNA metabolism in processes such as replication and damage response. Phosphorylated RPA32 (pRPA32) binds to RPA70 and possibly regulates the transient RPA70-Bloom syndrome helicase (BLM) interaction to inhibit DNA resection. However, the structural details and determinants of the phosphorylated RPA32–RPA70 interaction are still unknown. In this study, we provide molecular details of the interaction between RPA70 and a mimic of phosphorylated RPA32 (pmRPA32) using fluorescence polarization and NMR analysis. We show that the N-terminal domain of RPA70 (RPA70N) specifically participates in pmRPA32 binding, whereas the unphosphorylated RPA32 does not bind to RPA70N. Our NMR data revealed that RPA70N binds pmRPA32 using a basic cleft region. We also show that at least 6 negatively charged residues of pmRPA32 are required for RPA70N binding. By introducing alanine mutations into hydrophobic positions of pmRPA32, we found potential points of contact between RPA70N and the N-terminal half of pmRPA32. We used this information to guide docking simulations that suggest the orientation of pmRPA32 in complex with RPA70N. Our study demonstrates detailed features of the domain-domain interaction between RPA70 and RPA32 upon phosphorylation. This result provides insight into how phosphorylation tunes transient bindings between RPA and its partners in DNA resection.

Replication protein A (RPA) is the major eukaryotic ssDNA-binding protein and is involved in many DNA metabolism processes (*e.g.* DNA replication and repair) (1, 2, 3). RPA consists of three subunits of 70, 32, and 14 kDa, as shown in Fig. 1. In these three subunits, RPA contains six oligonucleotide binding folds termed DNA-binding domain (DBD) A, B, C, D, E, and F. DBD A (RPA70A) is the main ssDNA-binding domain. DBD F is in the N-terminal region of the 70-kDa subunit and is also called the RPA70N domain. It is known to be involved in many protein-protein interactions (4, 5, 6, 7, 8). The proteins that interact with RPA70N include damage response proteins such as ATRIP, RAD9, p53, and MRE11 (4, 5). Also, RPA70N interacts with various helicases, including Bloom syndrome protein (BLM), Werner syndrome protein (WRN), and Fanconi anemia group J protein (FANCJ) (7, 8). Notably, it has been shown that RPA stimulates the helicase activities of BLM and WRN through their interaction with RPA70N. It was revealed that BLM lacking its RPA-interacting site showed reduced unfolding activity for long DNA (9). RPA-BLM interaction is mainly mediated by two acidic sequences in the N-terminal region (BLM_153–165_ and BLM_290–301_) (7, 9). In the presence of RPA, WRN showed unfolding activity with long DNA and became a “superhelicase” (10, 11). As shown previously, RPA70N contains a basic cleft region located between the L12 and L45 loops (7). L12 connects the first and second β sheets, and L45 is the loop between the fourth and fifth β sheets. Most protein-protein interactions mediated by RPA70N occur within this basic cleft region, which interacts with areas of partner proteins rich in acidic amino acids.

**Figure 1:**
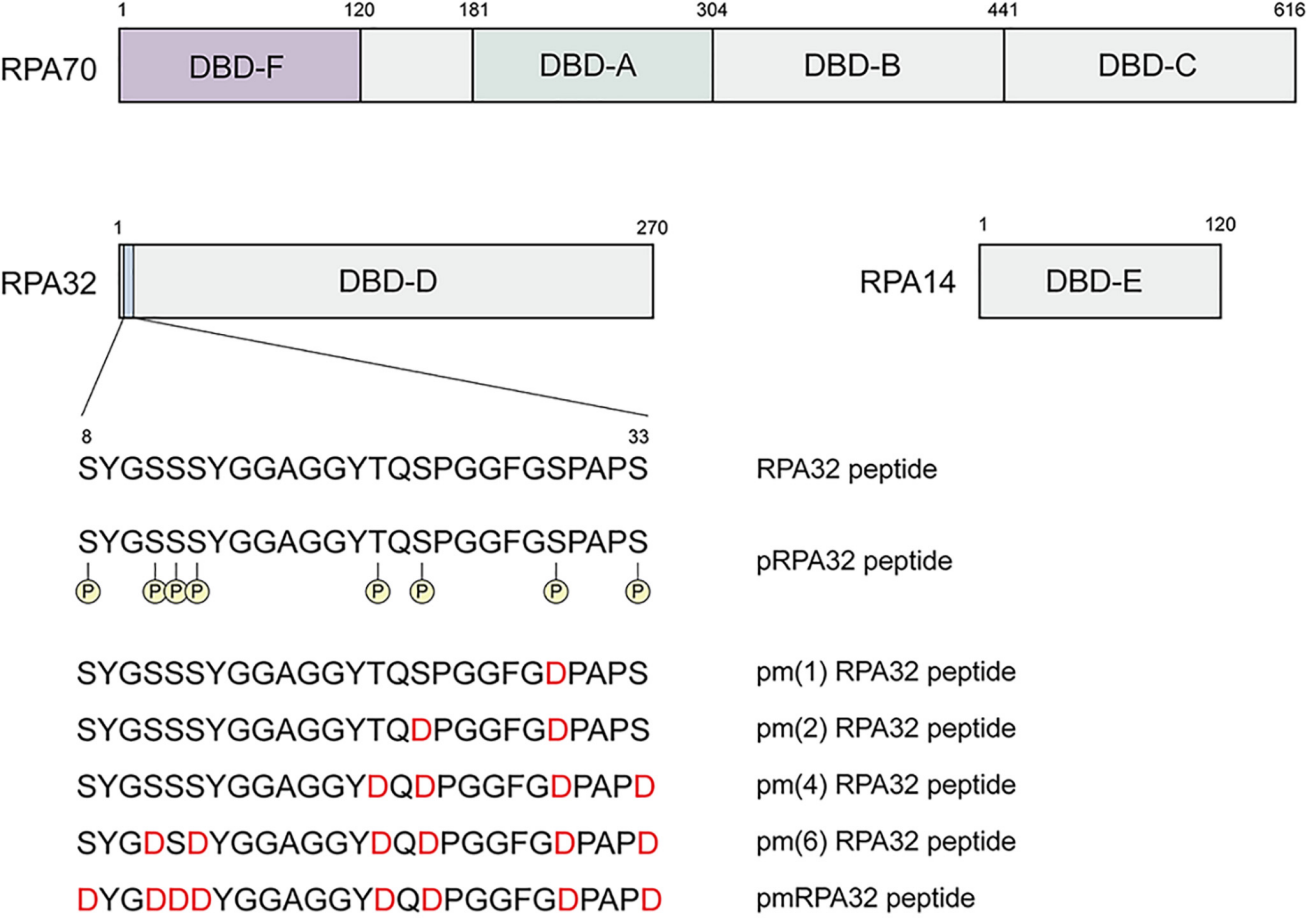
**Schematic drawing of RPA subunits and their domains.** RPA70 consists of DBD-F (RPA70N), DBD-A (RPA70A), DBD-B (RPA70B), and DBD-C (RPA70C). The potential phosphorylation sites in the N terminus of RPA32 are indicated. The amino acid sequences of the pm(1), pm(2), pm(4), and pm(6) RPA32 and pmRPA32 peptide are shown.

In addition to RPA70N, the phosphorylated RPA32 also plays a role in DNA metabolism via protein-protein interactions (12, 13, 14, 15, 16). There are eight potential phosphorylation sites in the N-terminal region of RPA32: Ser-8, Ser-11, Ser-12, Ser-13, Thr-21, Ser-23, Ser-29, and Ser-33 (Fig. 1) (12). CDC2 kinase and DNA-dependent protein kinase (DNA-PK) phosphorylate these sites in a cell cycle–dependent manner (13). Ser-29 is phosphorylated by CDC2, and Thr-21 and Ser-33 are phosphorylated by DNA-PK (13). In addition to kinases, these sites are phosphorylated by exposure to UV radiation. By UV-induced phosphorylation, Ser-11, Ser-12, Ser-13, Thr-21, Ser-23, Ser-29, and Ser-33 are phosphorylated even *in vitro* (14). It has been suggested that Ser-8 is phosphorylated *in vivo* after UV irradiation (14). Furthermore, RPA-ssDNA filaments can be phosphorylated by ataxia telangiectasia Rad3-related protein, cyclin-dependent kinase, and DNA-PK's catalytic subunit (15, 16). It has been shown that the phosphorylation of Ser-23, Ser-29, and Ser-33 are prerequisite for further phosphorylation of other sites, such as Ser-8 and Thr-21 (16).

In a recent study (17), it was revealed that RPA32 phosphorylation affects the DNA resection process by possibly regulating the transient binding of RPA70N to BLM. BLM is complexed with an exonuclease, either EXO1 or DNA2 (18). BLM/EXO1 resects one strand of DNA after double-strand breaks and generates a long 3′ ssDNA tail (19). WT RPA enhances the BLM's processivity by suppressing the strand-switching activity of BLM via RPA70N–BLM interaction. However, phosphorylated and phosphomimetic mutant RPA32 (pmRPA32) do not suppress the strand-switching activity of BLM. RPA70N likely interacts with phosphorylated RPA32 instead of BLM. In this way, it results in inhibition of DNA resection (17).

A brief NMR study has been performed on the interaction of pmRPA32 and RPA70N (20). However, the structural details and determinants of the interaction, including the physical basis of the inhibition of DNA resection by phosphorylated RPA, are still unknown. To better understand the interaction of phosphorylated RPA32 and RPA70N and to study whether the simple displacement of BLM from RPA70N is mediated by the phosphorylated or pmRPA32, we employed fluorescence polarization anisotropy (FPA) and NMR spectroscopy. We found that the pmRPA32 peptide specifically binds to RPA70N via electrostatic and hydrophobic interaction. We measured the dissociation constants for partial phosphomimetic constructs and the alanine mutants of hydrophobic residues in pmRPA32. Also, we determined the specific interface on RPA70N for the two tyrosine residues in pmRPA32. Based on the experimental results, we performed molecular docking simulations for the pmRPA32–RPA70N complex. Our study provides detailed information on the binding determinants of the interaction between RPA70 and phosphorylated RPA32.

## Results

### pmRPA32 peptide specifically binds to the basic cleft of RPA70N

We mimicked the phosphorylation of RPA32 by introducing aspartic acids into the phosphorylation sites (Fig. 1). This technique has been commonly used to mimic phosphorylation (20, 21), and the pmRPA32 construct showed similar properties to phosphorylated RPA32 in terms of inhibition of DNA resection in a previous study (17). Based on this, we assumed that this approach is valid to study the effects of RPA32 phosphorylation for RPA70N binding.

**Figure 2:**
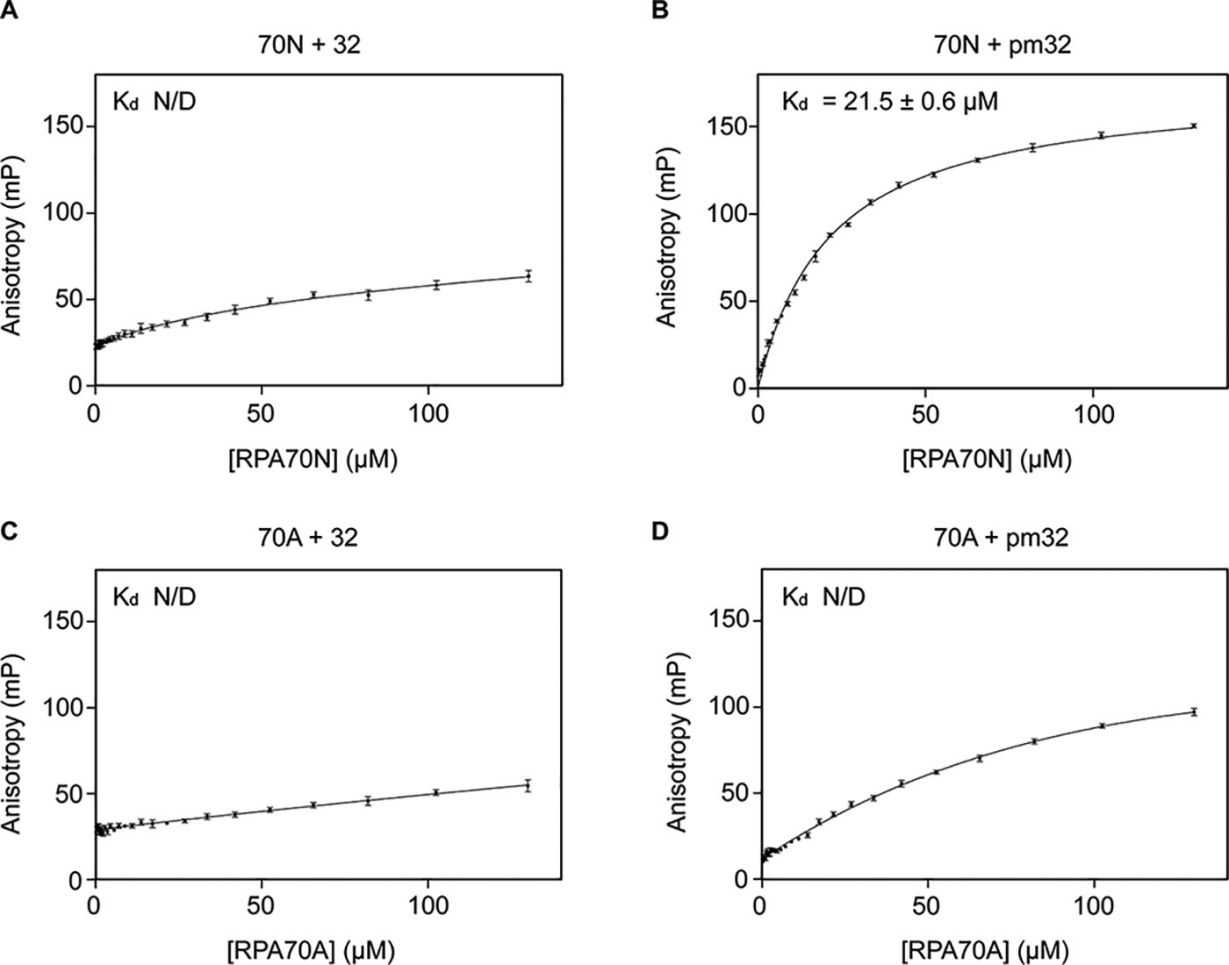
**Fluorescence polarization anisotropy of each combination.***A*, FITC-labeled RPA32 peptide + RPA70N. *B*, FITC-labeled pmRPA32 peptide + RPA70N. *C*, FITC-labeled RPA32 peptide + RPA70A. *D*, FITC-labeled pmRPA32 peptide + RPA70A. *K_d_* values are shown in each *panel*. *N/D*, not determined.

Fig. 2 shows the result of FPA assays of the FITC-labeled RPA32 and pmRPA32 peptides with either RPA70N or RPA70A. It is known that the RPA70A also interacts with proteins such as Rad51 and WRN (8, 22). For this reason, we tested RPA70A for RPA32 binding as well as 70N. FITC, a widely used fluorescent dye, has been used to study RPA70N–peptide binding (7). It is known that FITC does not change the binding mode or binding affinity of peptides significantly (5). Among the tested constructs, only RPA70N with the pmRPA32 peptide showed a typical hyperbolic binding curve and was fitted to the equation shown under “Experimental procedures.” The *K_d_* value was estimated as 21.5 ± 0.6 μm. In the other three cases, the anisotropy values were not saturated with increasing concentrations of RPA70N or RPA70A and were not able to be fitted. These results indicate that only the pmRPA32 peptide binds to RPA70N, and it does not bind to RPA70A, whereas the original, unphosphorylated RPA32 peptide binds to neither 70A nor 70N.

**Figure 3:**
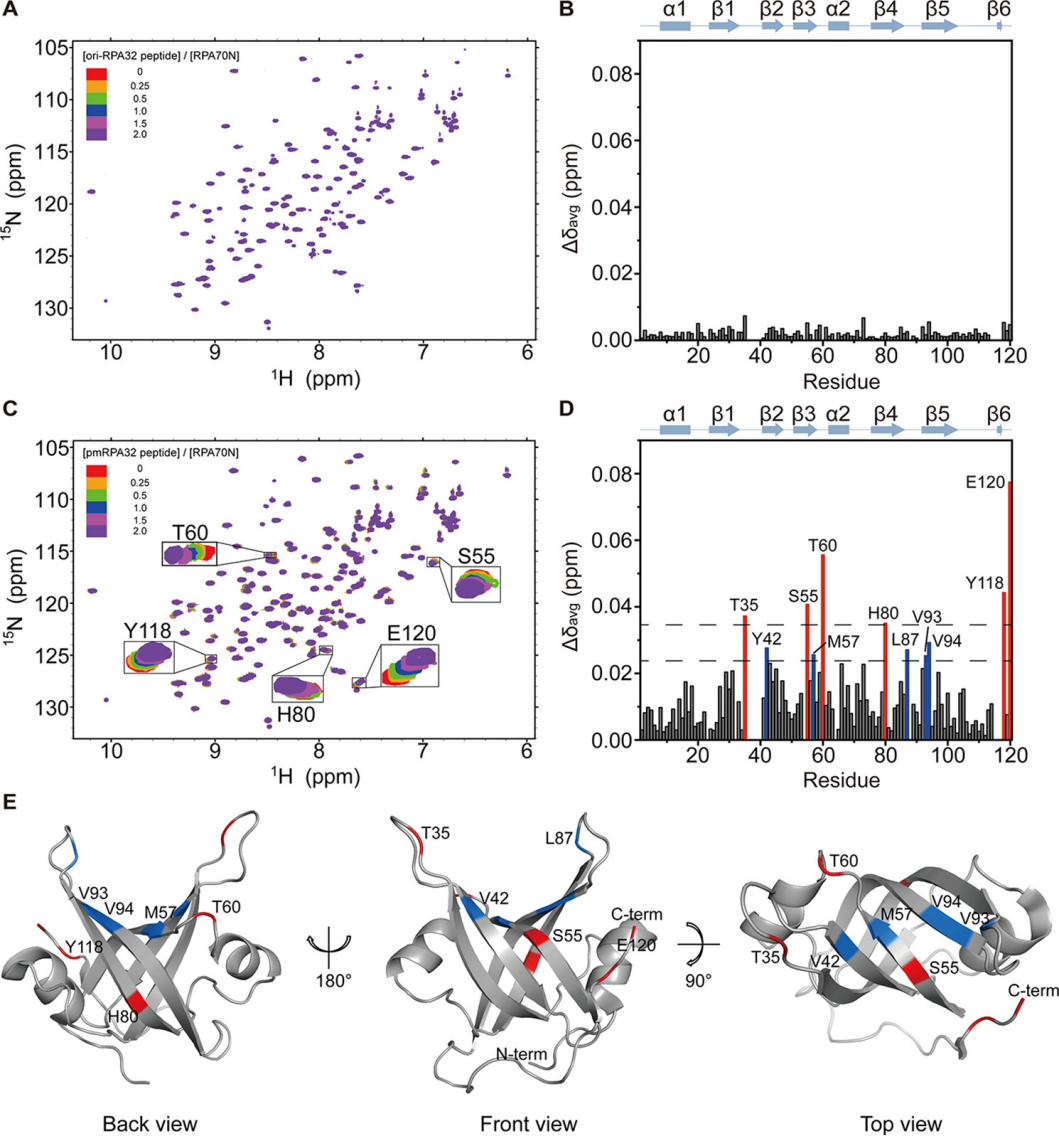
**NMR spectra of each combination.***A*, overlaid ^1^H–^15^N HSQC spectra of ^15^N-labeled RPA70N at increasing molar ratios of the RPA32 peptide. *B*, chemical shift perturbations (Δδ_avg_) of ^15^N-labeled RPA70N induced by 2.0 eq of RPA32 peptide. *C*, overlaid ^1^H–^15^N HSQC spectra of ^15^N-labeled RPA70N at increasing molar ratios of the pmRPA32 peptide. *D*, chemical shift perturbations (Δδ_avg_) of ^15^N-labeled RPA70N induced by 2.0 eq of the pmRPA32 peptide. The *dotted lines* indicate one (*lower*) or two (*upper*) S.D. from the average. The residues with Δδ_avg_ value greater than two S.D. from average are *colored* in *red*, and Δδ_avg_ values greater than one S.D. are *colored* in *blue*. *E*, mapping of residues affected by pmRPA32 peptide binding on the structure of RPA70N (PDB entry 2B29) (23). Residues perturbed by at least two S.D. above the average are shown in *red*, and those perturbed by at least one S.D. above the average are shown in *blue*.

^1^H–^15^N HSQC titration experiments identified the pmRPA32-binding surface on RPA70N. Fig. 3*A* shows the overlaid ^1^H–^15^N HSQC spectra of ^15^N-labeled RPA70N during titration with the original RPA32 peptide. Most of the cross-peaks were not perturbed at all. Fig. 3*B* quantifies the Δδ_avg_ of each residue. All Δδ_avg_ values were <0.01, which means that the chemical shift perturbation was barely observed. This is consistent with the FPA results showing that the unphosphorylated RPA32 peptide does not bind to RPA70N. Fig. 3*C* shows the superimposition of ^1^H–^15^N HSQC spectra of RPA70N during titration with the pmRPA32 peptide. Several residues, including Ser-55 and Thr-60, showed significant chemical shift perturbations upon pmRPA32 addition. The per-residue Δδ_avg_ values are shown in Fig. 3*D*. Thr-35, Ser-55, Thr-60, His-80, Tyr-118, and Glu-120 showed Δδ_avg_ values of two S.D. above the average, and Tyr-42, Met-57, Leu-87, Val-93, and Val-94 showed Δδ_avg_ values of one S.D. above the average. Compared with previous studies investigating binding between RPA70N and acidic peptides from various proteins (4, 5, 6, 7, 8), it is distinctive that His-80 showed significant chemical shift perturbation with pmRPA32. The significantly perturbed residues are mapped on the three-dimensional structure of RPA70N in Fig. 3*E* (PDB code 2B29) (23). Most of the perturbed residues are clustered near the L12 and L45 loops and the basic cleft region. Tyr-118 and Glu-120 are located in the C-terminal unstructured region, so we assume that perturbation of those residues could be allosteric. His-80 is in the β4 strand, located on the “backside” of RPA70N (*back view* of Fig. 3*E*). We assume that His-80 is perturbed because of the length of the pmRPA32 peptide, which is 26 amino acids. Compared with previous studies (4, 5, 6, 7, 8), pmRPA32 is longer than other acidic peptides that bind to RPA70N, generally 11–15 amino acids (Table 1). Thus, His-80 of RPA70N could be on the binding surface for the longer pmRPA32 peptide compared with other RPA70N-binding peptides. Taken together, we showed that the pmRPA32 peptide binds to the basic cleft region of RPA70N and that His-80 additionally participates in the pmRPA32 binding.

**Table 1:** Comparison of the RPA70-binding sequences

	No. of amino acids	No. of acidic amino acids	Net charge	*K_d_*
				μ*m*
pmRPA32_8–33_	26	8	−8	21.5 ± 0.6
pm(6) RPA32	26	6	−6	24.7 ± 1.0
BLM_153–165_ (7)	13	7	−7	5.76 ± 0.86
BLM_290–301_ (7)	12	7	−7	13.5 ± 2.4
WRN_435–450_ (8)	16	6	−5	41.4 ± 3.3
FANCJ_1120–1133_ (8)	14	6	−6	40.2 ± 1.8
ETAA1_900–912_ (6)	13	6	−6	NA^a^
ATRIP_54–68_ (5)	15	6	−6	28.6 ± 3.1
RAD9_297–311_ (5)	15	5	−5	51.4 ± 8.9
p53_44–58_ (5)	15	5	−5	99.9 ± 8.4
MRE11_539–553_ (5)	15	4	−4	65.8 ± 23.7


a Not reported in previous research.

### At least six phosphomimetic substitutions are necessary for RP70N binding

Our results clearly showed that the full pmRPA32 peptide, which contains eight aspartic acids, specifically binds to RPA70N. Because there is interdependence between the phosphorylation sites on RPA32, each phosphorylation site could affect RPA70N binding differently. To investigate the effects of each site and to reveal the required minimum number of acidic amino acids, we tested partial pmRPA32 peptides for RPA70N binding.

**Figure 4:**
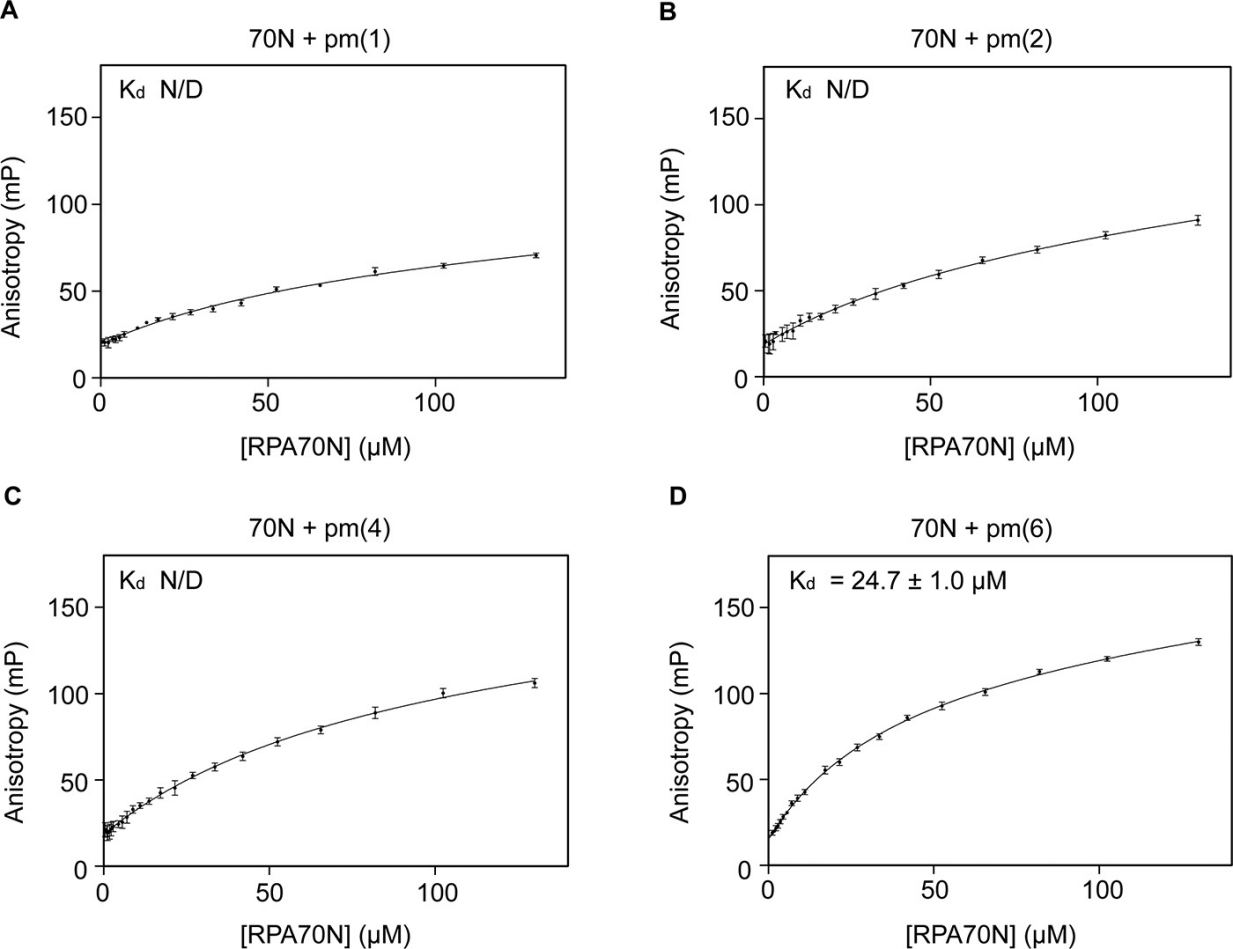
**Fluorescence polarization anisotropy of each combination.***A*, FITC-labeled pm(1) peptide + RPA70N. *B*, FITC-labeled pm(2) peptide + RPA70N. *C*, FITC-labeled pm(4) peptide + RPA70N. *D*, FITC-labeled pm(6) peptide + RPA70N. *K_d_* values are shown in each *panel*. *N/D*, not determined.

We designed four kinds of peptides with different amounts of negative charge. For pm(1), only Ser-29 was substituted with Asp. We additionally replaced Ser-23 with Asp for pm(2). The phosphorylation of these residues is required for further phosphorylation of other sites on RPA32 (16). T21D and S33D were further changed for pm(4). Finally, for pm(6), Ser-11 and Ser-13 were additionally replaced by Asp (12). We performed FPA assays for each peptide and RPA70N. For pm(1), pm(2), and pm(4), the anisotropy did not fit the model correctly (Fig. 4, *A–C*). For pm(6), the data fit well to the “one site–specific” model with a good correlation coefficient, *R*^2^ ≥ 0.96 (Fig. 4*D*). The calculated *K_d_* value for pm(6) was 24.7 ± 1.0 μm, which is comparable with that of the fully substituted peptide. Thus, phosphomimetic peptides of prerequisite sites (pm(1), pm(2), and pm(4)) could not bind to RPA70N, and this implies that at least six substitutions of the RPA32 peptide are required for RPA70N binding.

### The aromatic amino acids Tyr-9 and Tyr-14 are required for RPA70N binding

**Figure 5:**
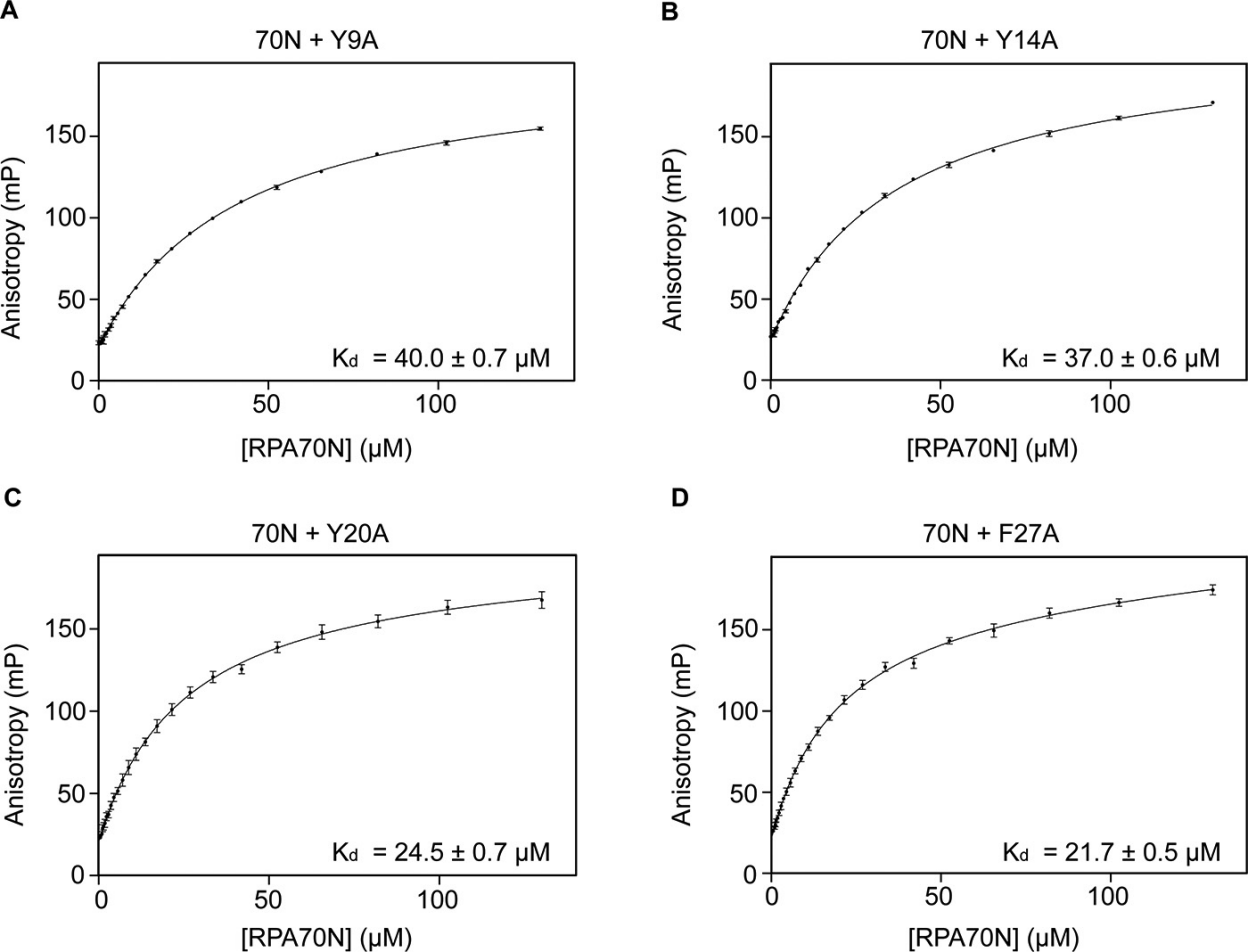
**Fluorescence polarization anisotropy of each combination.***A*, FITC-labeled pmRPA32-Y9A peptide + RPA70N. *B*, FITC-labeled pmRPA32-Y14A peptide + RPA70N. *C*, FITC-labeled pmRPA32-Y20A peptide + RPA70N. *D*, FITC-labeled pmRPA32-F27A peptide + RPA70N. *K_d_* values are shown in each *panel*.

Previous studies of RPA70N interaction with various acidic peptides showed that hydrophobic interactions, as well as electrostatic interactions, mediate complex formation (4, 5, 6, 7, 8) (Fig. S1). To investigate the effects of aromatic amino acids in the pmRPA32 peptide on RPA70N binding, we designed four mutants that replace each hydrophobic residue with alanine (Y9A, Y14A, Y20A, and F27A). We performed FPA assays with RPA70N (Fig. 5). Increasing concentrations of RPA70N were added to a solution of FITC-labeled pmRPA32 peptide. The *K_d_* value was estimated as 40.0 ± 0.7 μm for Y9A, 37.0 ± 0.6 μm for Y14A, 24.5 ± 0.7 μm for Y20A, and 21.7 ± 0.5 μm for F27A. Compared with the pmRPA32 peptide, Y9A and Y14A had 2-fold less affinity, but Y20A and F27A maintained similar binding affinities. Our results suggest that Tyr-9 and Tyr-14 are more important for RPA70N binding, whereas Tyr-20 and Phe-27 do not participate in the interaction. This implies that the aromatic amino acids in the N-terminal half of the pmRPA32 peptide could contribute to RPA70N binding, along with phosphorylation.

**Figure 6:**
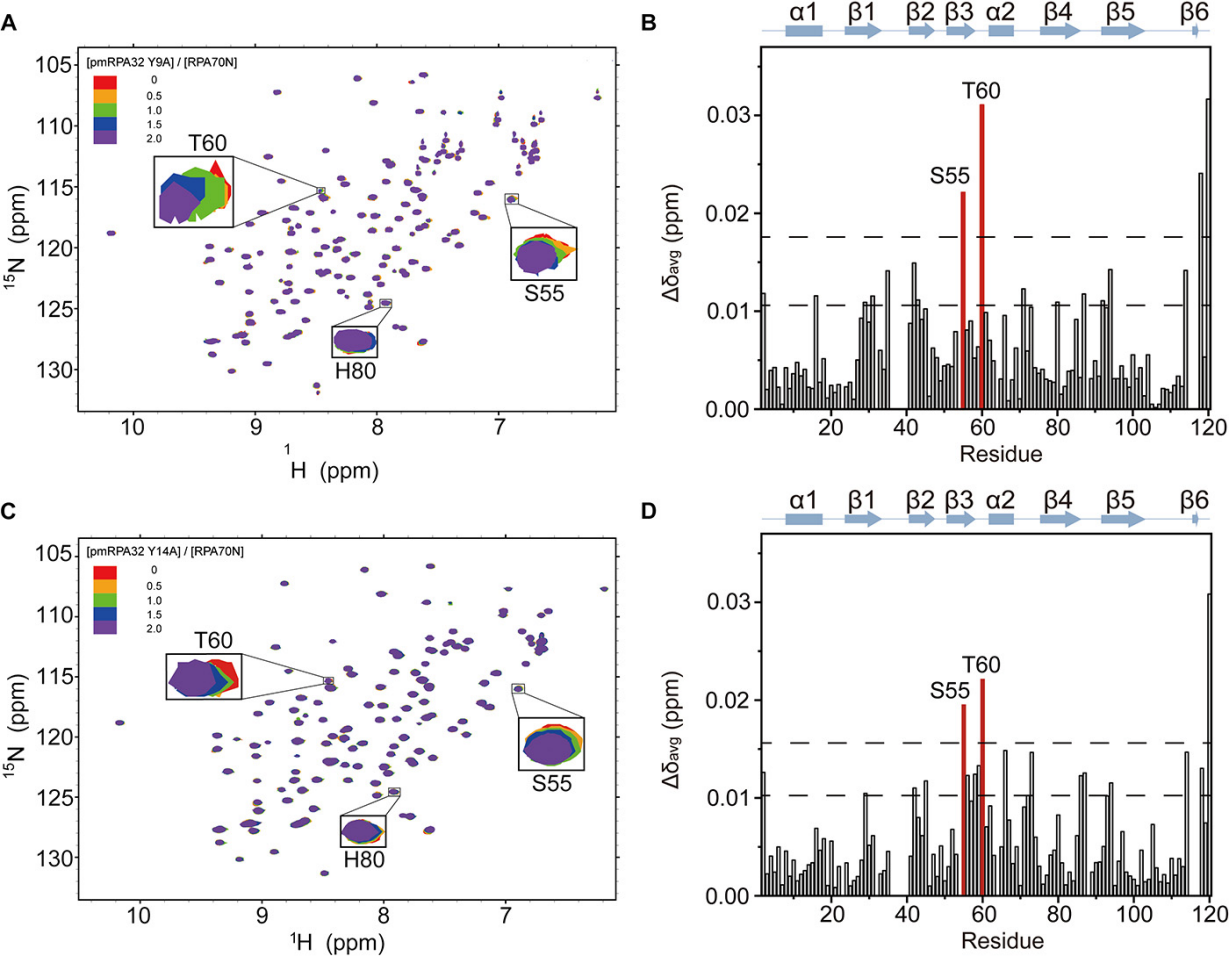
**NMR spectra of RPA70N with pmRPA32-Y9A and -Y14A peptides.***A*, overlaid ^1^H–^15^N HSQC spectra of ^15^N-labeled RPA70N at increasing molar ratios of pmRPA32-Y9A peptide. *B*, chemical shift perturbations (Δδ_avg_) of ^15^N-labeled RPA70N induced by 2.0 eq of pmRPA32-Y9A peptide. The *upper dotted line* indicates two S.D. from the average, and residues with values above this threshold (except the terminal region) are *colored* in *red*. *C*, overlaid ^1^H–^15^N HSQC spectra of ^15^N-labeled RPA70N at increasing molar ratios of pmRPA32-Y14A peptide. *D*, chemical shift perturbations (Δδ_avg_) of ^15^N-labeled RPA70N induced by 2.0 eq of pmRPA32-Y14A peptide. The *color scheme* is the same as in *B*.

To identify the interacting surface of RPA70N with Tyr-9 and Tyr-14 of the pmRPA32 peptide, we titrated Y9A and Y14A into ^15^N-labeled RPA70N and performed ^1^H–^15^N HSQC titration experiments. Fig. 6 (*A* and *C*) shows the superimposed ^1^H–^15^N HSQC spectra of RPA70N upon the addition of Y9A and Y14A peptides, respectively. In both cases, Ser-55 and Thr-60 showed sizeable chemical shift perturbations as each peptide was added. Fig. 6 (*B* and *D*) show the chemical shift changes of RPA70N upon the addition of Y9A and Y14A peptides, respectively. In both cases, Ser-55, Thr-60, and Glu-120 showed Δδ_avg_ values of at least two S.D. above the average. Compared with the titrations of pmRPA32 (Fig. 3*D*), all chemical shift changes were reduced by about half on average in both titrations. This is consistent with the weakened binding affinity measured in FP assays. Among the three significantly perturbed residues of RPA70N by the pmRPA32 peptide, Ser-55 and Thr-60 still showed significant chemical shift perturbations of larger than two S.D. from the average upon Y9A and Y14A titration. However, His-80 of RPA70N did not show considerable chemical shift change in either titration. This suggests that His-80 of RPA70N could make contact with Tyr-9 or Tyr-14 of pmRPA32, and the alanine mutation of either residue eliminates this interaction.

### Docking models suggest that His-80 of RPA70N could be a contact point for either Tyr-9 or Tyr-14 of pmRPA32

**Figure 7:**
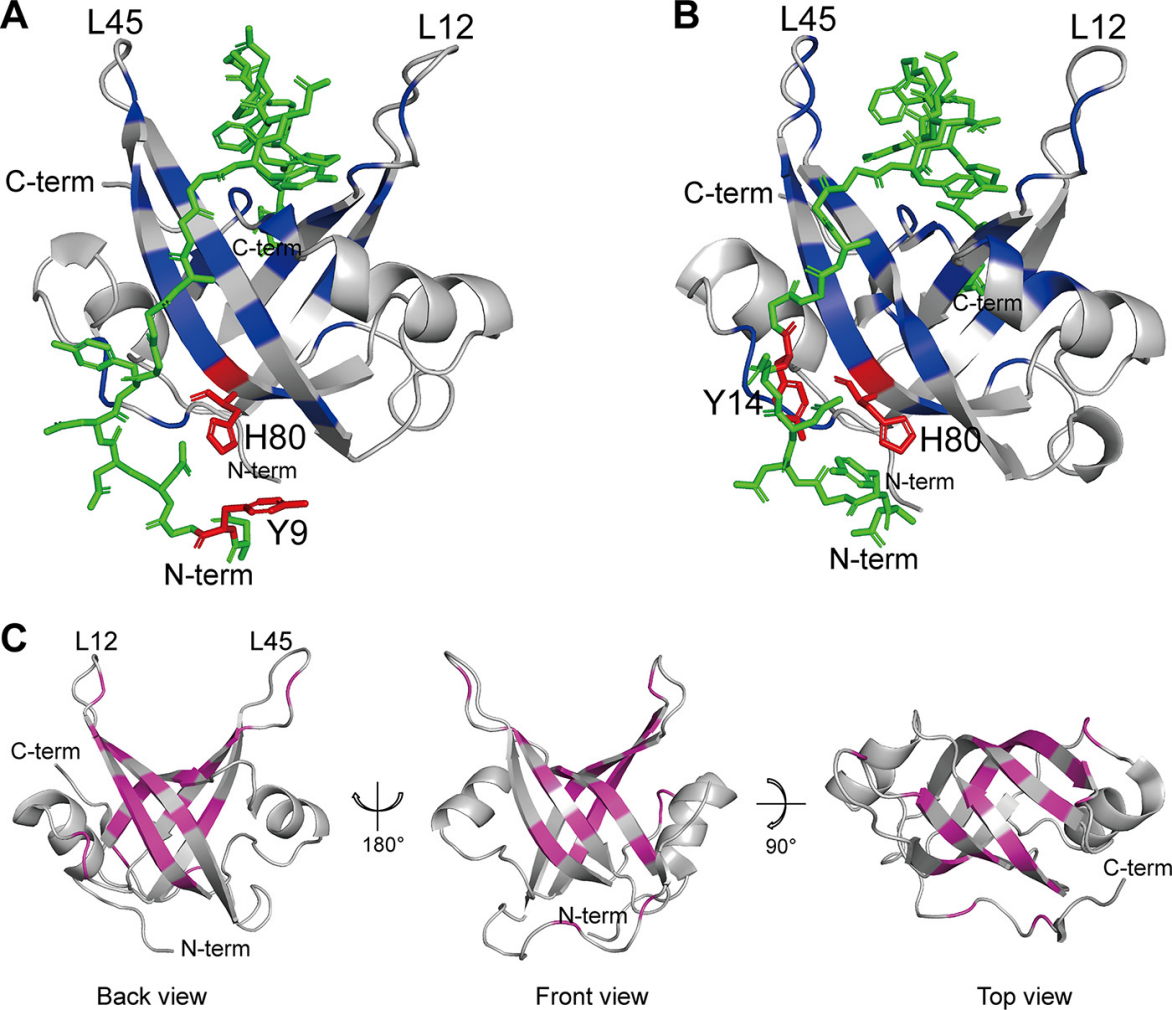
**Docking model structures of the RPA70N–pmRPA32 peptide complex from the major cluster.***A*, docking model structure with RPA70N His-80–pmRPA32 Tyr-9 as the contact pair. *B*, docking model structure with RPA70N His-80–pmRPA32 Tyr-14 as the contact pair. The pmRPA32 peptide is displayed in *green*. RPA70N His-80–pmRPA32 Tyr-9 (*A*) and RPA70N His-80–pmRPA32 Tyr-14 (*B*) contact pairs are *colored* in *red*. The residues within 4.5 Å of the pmRPA32 peptide are shown in *blue*. *C*, mapping of the overlapped interfaces given the restriction of His-80–Tyr-9 and Tyr-14 on the structure of RPA70N (PDB code 2B29) (22). The residues are *colored* in *pink*.

To further investigate and visualize RPA70N's interaction with the pmRPA32 peptide, docking simulations were performed with the CABS-dock Web server (24). Based on the ^1^H-^15^N HSQC titration experiments of the Y9A and Y14A peptides into RPA70N, we performed two independent docking simulations, using either RPA70N His-80–pmRPA32 Tyr-9 or RPA70N His-80–pmRPA32 Tyr-14 as the contact point. For each simulation, 1,000 models were generated and classified into 10 clusters. The major cluster (cluster 1) from each simulation had more than twice the cluster density of the other nine clusters. For this reason, cluster 1 from each simulation was chosen as the representative structure. Fig. 7 shows the reconstructions with minimal atomic energy of the RPA70N–pmRPA32 peptide complex, with RPA70N His-80–pmRPA32 Tyr-9 (Fig. 7*A*) and RPA70N His-80–pmRPA32 Tyr-14 (Fig. 7*B*) as restraints. In both models, the pmRPA32 peptides are in an extended conformation, consistent with CD data showing that pmRPA32 does not have any secondary structure and that RPA70N binding does not induce any secondary structure (Fig. S2). The major clusters showed the basic cleft region of RPA70N as the binding surface of the pmRPA32 peptide. In the representative model structure based on the RPA70N His-80–pmRPA32 Tyr-9 restraint, Thr-19, Asn-20, Lys-22, Ile-28, Asn-29, Arg-31, Ser-38, Arg-41, Arg-43, Leu-45, Thr-52, Ser-54, Ser-55, Met-57, Gln-78, His-80, Arg-81, Phe-82, Ile-83, Asn-85, Leu-87, Val-93, Ile-95, Met-97, Glu-100, Lys-111, Asn-114, Pro-115, and Pro-117 of RPA70N are close to the pmRPA32 peptide (<4.5 Å) (Fig. 7*A*). Similar surfaces, including Thr-19, Asn-20, Ile-21, Lys-22, Ile-28, Asn-29, Ser-38, Pro-39, Arg-41, Arg-43, Leu-45, Thr-52, Ser-54, Ser-55, Met-57, Thr-60, Asn-63, Gln-78, His-80, Arg-81, Phe-82, Ile-83, Asn-85, Leu-87, Asp-89, Arg-91, Val-93, Ile-95, Met-97, Glu-98, Lys-111, and Pro-115 are located within 4.5 Å of the peptide when using the RPA70N His-80–pmRPA32 Tyr-14 restraint (Fig. 7*B*). Among these residues, Ser-55, Met-57, His-80, Leu-87, and Val-93 showed large chemical shift perturbations in our ^1^H-^15^N HSQC experiments. Other amino acids are also located near the peptide, which showed large chemical shift perturbations. The peptide orientations in both simulations were the same, with the N terminus of the peptide heading “backward” (*back view* of Fig. 3*E*) and the C terminus heading “forward” (*front view* of Fig. 3*E*) with respect to the basic cleft of RPA70N. Remarkably, more than 75% of the peptide interface on RPA70N overlapped in the two cases (Fig. 7*C*). This implies that the contact of His-80 of RPA70N with either Tyr-9 or Tyr-14 of pmRPA32 results in a similar binding interface. The details of the structural clustering of each model and stereo images of cluster 1 from each simulation are shown in the supporting information (see Table S1 and Fig. S3).

## Discussion

In this study, we confirmed that a peptide mimicking the phosphorylated N terminus of RPA32 (aa 8–33) binds specifically to RPA70N. Our FPA data showed that the pmRPA32 peptide has a binding preference for RPA70N over RPA70A. Also, the binding affinity of the unphosphorylated RPA32 peptide sequence to RPA70N is negligible. With this information, it can be deduced that phosphorylation at the N terminus of RPA32 would induce the interaction of pRPA32 and RPA70N. This interaction occurs using the basic cleft region of RPA70N, as indicated by NMR analysis. Similar to previous studies (7, 8), this basic cleft region of RPA70N is the binding interface for pmRPA32. As it consists of basic amino acids, acidic residues of the peptide should play an essential role in the interaction—in this case aspartic acids, which mimic phosphorylation. By comparison with other RPA70N-binding peptides (Table 1 and Fig. S1), we suggest two significant determinants for RPA70N binding.

The first determinant is sufficient numbers of acidic amino acids. By the FPA experiment, we revealed that six aspartic acids were needed to provide enough negative charge for binding with the basic cleft of RPA70N. For the pm(6) peptide, the net charge value is −6, and −5 to −6 is also predominant in the identified RPA70N-binding sequences (Table 1). In a previous study, the basic cleft site containing Ser-55 and Thr-60 was hydrophobic, with positively charged residues Arg-31, Arg-43, and Arg-91 in proximity to Ser-55 (25). This suggests that enough negative charge of binding peptides could favorably interact with positively charged surfaces via charge-charge interactions. Our data show that four acidic amino acids are not sufficient for RPA70 binding. Based on this, we suggest that at least six acidic residues are required for RPA70N binding.

The second determinant of the interaction is the presence of aromatic amino acids on the peptides. Fig. S1 shows that all known peptide binders of RPA70N except p53 have an aromatic amino acid at the second position from the N terminus of the sequence. Furthermore, several peptide binders contain additional aromatic amino acids. The importance of the aromatic amino acid at the C terminus of FANCJ_1120-1133_ for RPA70N binding was previously revealed by a point mutation experiment (8). In this study, we showed that the alanine mutation of Tyr-9 and Tyr-14 significantly reduced the binding affinity. Therefore, we suggest that both a sufficient number of acidic amino acids and aromatic amino acids at strategic positions are required for RPA70N interaction.

By comparing chemical shift perturbation data of Y9A and Y14A with that of pmRPA32, we found that His-80 of RPA70N was less perturbed by the mutant peptides. This indicates that His-80 of RPA70N initially makes contact with Tyr-9 or Tyr-14 of the pmRPA32 peptide. As we mentioned above, His-80 of RPA70N is a unique interface for pmRPA32, whereas Ser-55 and Thr-60 are common binding surfaces of several RPA70N binders (4, 5, 6, 7, 8). It is suspected that either Tyr-9 or Tyr-14 could contact His-80 of RPA70N because the pmRPA32 peptide does not have a rigid secondary structure. Our data imply that the N-terminal region of pmRPA32 is close to the “backside” of RPA70N, and the remaining region makes contact with binding surfaces containing Ser-55 and Thr-60.

These results provided information on the residue-level contacts between RPA70N and pmRPA32, which were used to visualize the complex by molecular docking simulations. Because docking simulations of RPA70N–pmRPA32 without any restraints showed mixed orientations of the peptide in the complex (data not shown), we used the hydrophobic pairings as contact points for determining the orientation of the pmRPA32 peptide in the complex. The resulting docking models have the same orientation of the peptide. In each model, Tyr-9 or Tyr-14 of the pmRPA32 peptide interacts with His-80 of RPA70N. This indicates that the pmRPA32 N terminus is on the “backside” near RPA70N His-80 and that the pmRPA32 C terminus region is on the “front side.” This orientation is clearly distinguishable because the His-80 and β4 region of RPA70N is only perturbed in the current study (4, 5, 6, 7, 8). Therefore, His-80 may be considered as a new intervention point for developing RPA70N inhibitors.

As we mentioned above, phosphorylated and phosphomimetic RPA32 mutants inhibit DNA resection via disrupting the BLM-RPA70N interaction (17). However, the *K_d_* that we measured in this study for the pmRPA32–RPA70N interaction is higher than those of BLM–RPA70N interactions (5.76 ± 0.86 μm for BLM_153-165_ and 13.5 ± 2.4 μm for BLM_290-301_) measured with the same method (Table 1) (7). This implies that the phosphorylated and phosphomimetic RPA32 does not displace BLM on RPA70N based on a simple binding affinity difference, unlike p53 (23). To verify this, we performed an FPA competition assay with FITC-labeled BLM peptide and unlabeled pmRPA32 peptide to RPA70N, and the pmRPA32 peptide did not compete with BLM peptide (Fig. S4). Thus, we suggest that other factors, such as the whole RPA heterotrimer experiencing the conformational change upon RPA32 phosphorylation, are required for favorable pRPA32–RPA70N interaction. This is supported by a previous study, which showed that hyperphosphorylation at the N terminus of RPA32 induces a conformational change in RPA70 (26).

Previous studies emphasized that a binding affinity in the micromolar to millimolar range is vital for transient binding between proteins in cell-signaling and regulation processes (27, 28, 29). In this context, the *K_d_* values between RPA70N and the BLM peptides/pmRPA32 peptide, which are in the micromolar range, could facilitate the exchange of binding partners of RPA70N.

In summary, our study characterized the binding of pmRPA32 by RPA70N. By FPA experiments, we showed that the pmRPA32 peptide specifically binds to the RPA70N domain, not 70A, and when it is not phosphorylated, it binds to neither 70N nor 70A. We found that the N terminus of the pmRPA32 is involved in the formation of the RPA70N–pmRPA32 complex and that RPA70N H80 can be regarded as a new intervention point in that binding region. These data provide a new understanding of the interdomain interactions between RPA70 and RPA32, which is an essential step in DNA metabolism, especially DNA resection.

## Experimental procedures

### Sample preparation

RPA70N_1–120_ and RPA70A_181–304_ were expressed and purified as described previously (5, 30). ^15^N-Labeled RPA70N was also expressed and purified with the same method using M9 medium. All of the synthesized peptides except for the FITC-labeled peptide were purchased from Lugen Sci (Gyeonggi-do, Korea). FITC-labeled peptides were purchased from AnyGen (Gwangju, Korea). Purchased peptides were dissolved in 20 mm HEPES, 100 mm NaCl, 2 mm DTT, pH 7.4, buffer to make a 2 mm stock and stored at −20 °C until use.

### Fluorescence polarization anisotropy assay

In the previously performed FPA assay using ATRIP peptides and RPA70, it was confirmed that the FITC label does not affect the binding affinity significantly (5). For FITC labeling, a 6-aminocaproic acid (6-aminohexanoic acid) spacer was used. Increasing concentrations (0–150 μm) of RPA70N or RPA70A were prepared in an assay buffer of 20 mm HEPES, 100 mm NaCl, 2 mm DTT, pH 7.4, in Corning 96-well black flat-bottom plates (polystyrene, nontreated). To each well, FITC-RPA32 peptide was added to a final concentration of 50 nm, and the plates were incubated on a shaker for 1 h at 25 °C. The total volume of the reaction sample was 50 μl. The emission polarization anisotropy was calculated by referring to a previous publication (5). The fluorescence was measured using BioTek Cytation5 and GENE5 software (GIST, Gwangju) with excitation and emission wavelengths of 485 and 528 nm, respectively. GraphPad Prism version 7.01 (GraphPad Software, La Jolla, CA, USA) was used for emission anisotropy data visualization. Anisotropy values were plotted for each concentration of RPA70N and RPA70A. All experiments were repeated three times, and dissociation constants (*K_d_*) were calculated using the “one site–specific” fitting model of the GraphPad software. The equation used in the fitting is as follows,

(Eq. 1) $$y=\frac{B_{max}\times X}{K_{d}+X}$$

where y is the fluorescence anisotropy, *B*_max_ is the maximum specific binding, *K_d_* is the equilibrium binding constant, and *X* is the concentration of RPA. For the competitive FPA assay, increasing concentrations of the pmRPA32 peptide (0–250 μm) were added to 6 μm (for BLM_153-165_) or 13 μm (for BLM_290-301_) RPA70N and 500 nm FITC-labeled BLM peptides in 200 μl of assay buffer. Equilibrium fluorescence measurements were performed in the same way as described above. All experiments were repeated three times, and anisotropy values were plotted by the log concentrations of the pmRPA32 peptide. The “Log[inhibitor] *versus* response – variable slope (four-parameter)” model of GraphPad Prism version 7.01 was used for fitting and IC_50_ calculation. The equation is as follows,

(Eq. 2) $$y=bottom+\frac{\left(top-bottom\right)}{\left(1+10^{\left(\left({logIC}_{50}-x\right)\times Hillslope\right)}\right)}$$

where top and bottom terms are plateaus, and Hill slope represents the steepness of curves.

### NMR spectroscopy

NMR experiments were performed using a 700-MHz Bruker *AVANCE* II (KBSI, Ochang) and 600-MHz Bruker (Gwangju, GIST) spectrometer equipped with a cryoprobe at 298 K. NMR data were processed using Topspin software (Bruker) and analyzed with SPARKY software. ^15^N-Labeled RPA70N was prepared at 0.3 mm for each sample, and the peptide was added at the indicated molar ratios. All of the samples were prepared in 20 mm HEPES, 100 mm NaCl, 2 mm DTT, pH 7.4, buffer. The average chemical shift change (Δδ_avg_) was calculated by the following equation.

(Eq. 3) $$\Delta \delta_{avg}=\sqrt{{\left(\Delta \delta_{H}\right)}^{2}+{\left(\frac{\Delta \delta_{N}}{5.88}\right)}^{2}}$$

### Docking

The CABS-dock Web server was used for the docking simulations of RPA70N and pmRPA32 interaction (24). The crystal structure of RPA70N (PDB code 2B29) (23) was used for starting coordinates for the protein domain, and the coordinates for pmRPA32 were generated from the amino acid sequence. Fifty simulation cycles were used, and the contact pair (either RPA70N His-80–pmRPA32 Tyr-9 or RPA70N His-80–pmRPA32 Tyr-14) was constrained to within 4.5 Å. Detailed filtering and clustering methods were described previously (31). Docked models were visualized using PyMOL (32).

## Data availability

All data are contained within the article.
