# Supplementary material for: Determinants of replication protein A subunit interactions revealed using a phosphomimetic peptide
Source: J Biol Chem. 2021 Jan 13;295(52):18449–58. doi: 10.1074/jbc.RA120.016457 (PMC7939470; doi:10.1074/jbc.RA120.016457)

**Supplementary data**

**Figure S1.**

The aromatic amino acids (red) of the RPA70N binding sequences (4–8).

**
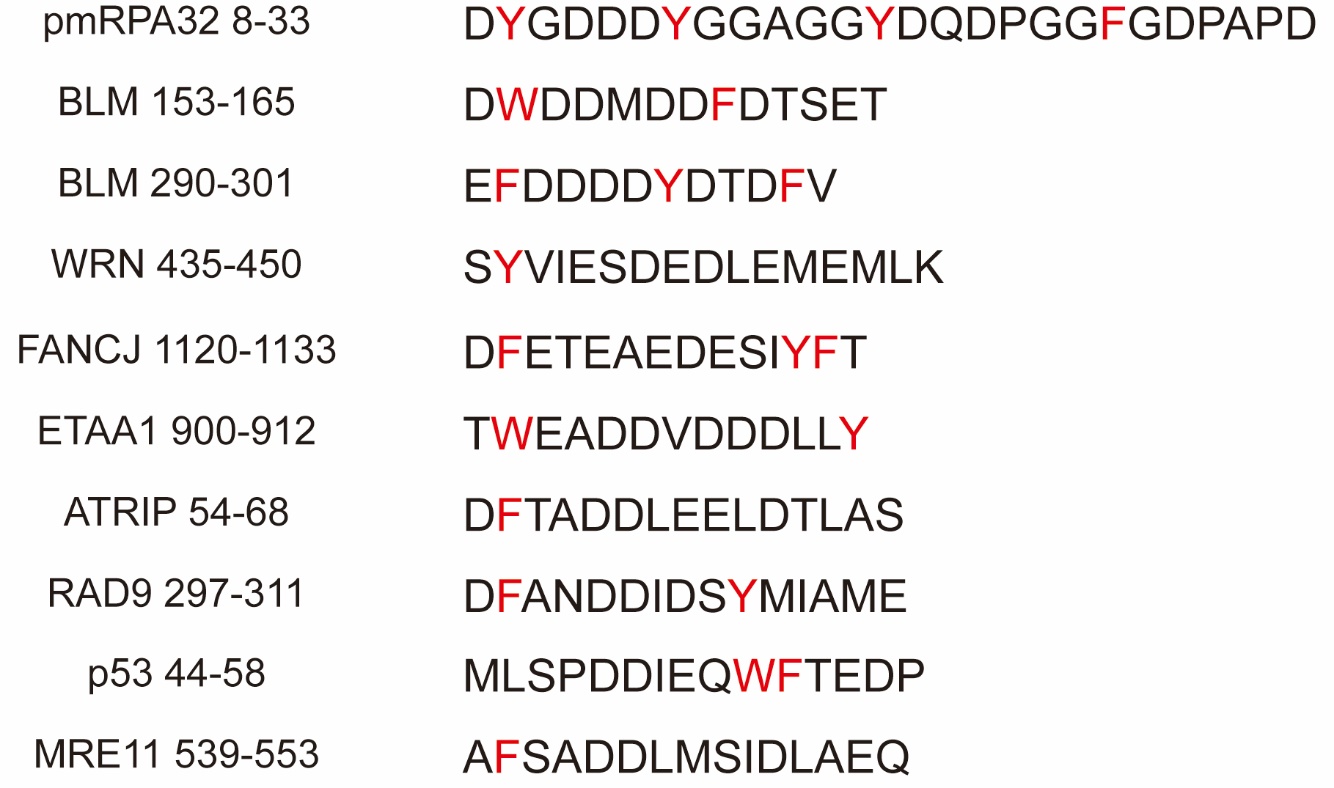
**

**Figure S2.**

CD spectra of RPA70N, pmRPA32 peptide and 70N – pmRPA32 complex.


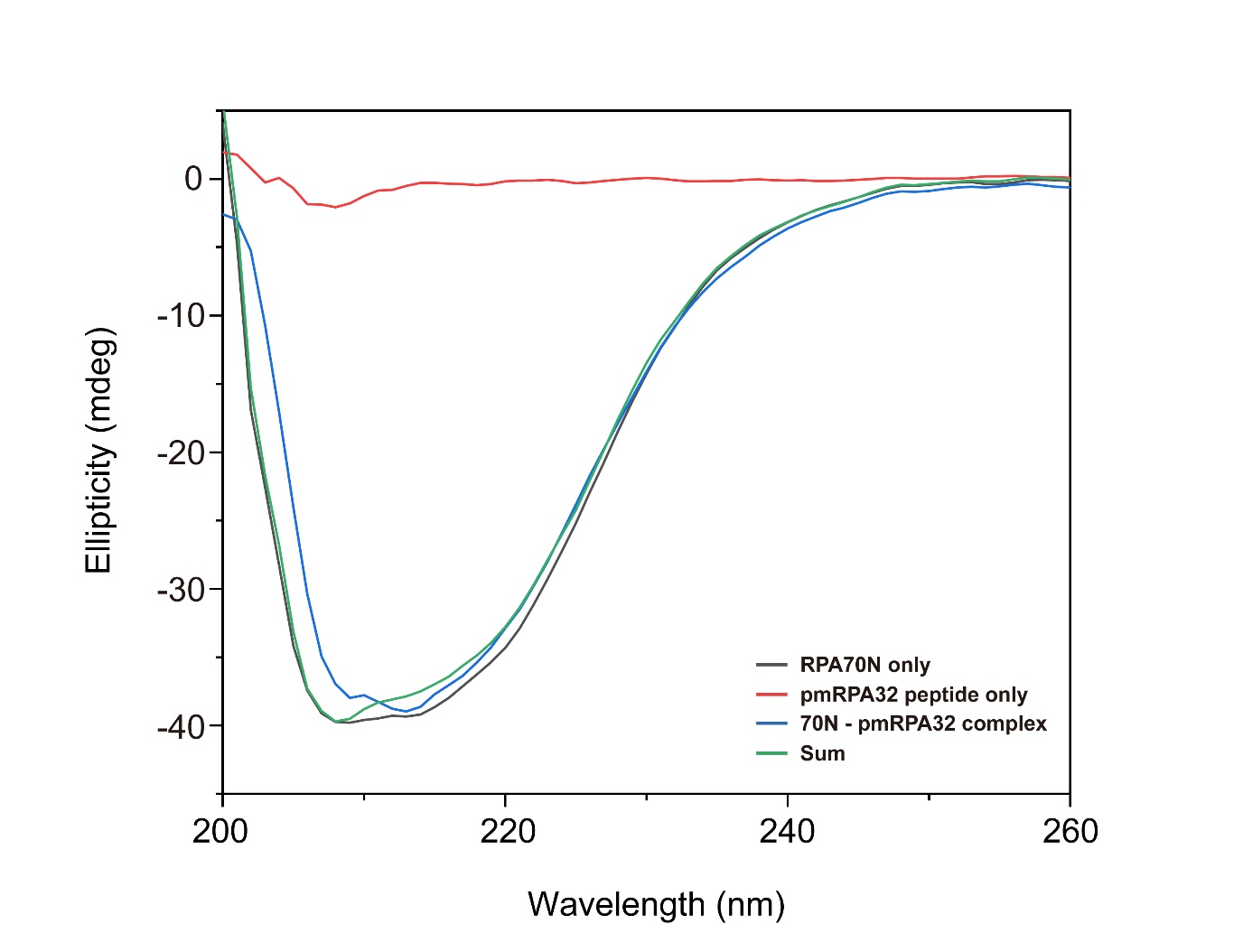


**Supplementary methods**

**CD spectroscopy**

CD spectra were observed using a JASCO J-815 CD spectrometer (GIST, Gwangju) at 20 ˚C. RPA70N or pmRPA32 peptide were dissolved in 20 mM HEPES, 100 mM NaCl, 2 mM DTT pH 7.4 buffer to a final concentration of 200 µM, and for the complex sample, pmRPA32 peptide was added to RPA70N with a molar ratio of 1:1 at a concentration 200 µM each. All the spectra were collected from 200 nm to 260 nm at a scanning speed of 100 nm/min and with a spectral bandwidth of 2 nm for each sample, and the spectra were observed after overnight incubation of samples.

**Table S1.**

Clustering details of docking.

|  |  | Cluster density | Average RMSD (Å) | Max RMSD (Å) | Numbers of elements |
| --- | --- | --- | --- | --- | --- |
| RPA70N H80 – pmRPA32 Y9 | Cluster 1 | 41.61 | 3.60 | 19.16 | 150 |
|  | Cluster 2 | 17.22 | 7.43 | 34.93 | 128 |
|  | Cluster 3 | 17.17 | 6.35 | 21.61 | 109 |
|  | Cluster 4 | 16.11 | 6.70 | 26.98 | 108 |
|  | Cluster 5 | 11.54 | 12.47 | 40.60 | 144 |
|  | Cluster 6 | 9.92 | 5.24 | 9.38 | 52 |
|  | Cluster 7 | 6.54 | 5.20 | 9.60 | 34 |
|  | Cluster 8 | 6.24 | 18.91 | 44.61 | 118 |
|  | Cluster 9 | 4.59 | 20.71 | 47.84 | 95 |
|  | Cluster 10 | 3.18 | 19.49 | 43.59 | 62 |
| RPA70N H80 – pmRPA32 Y14 | Cluster 1 | 34.59 | 5.35 | 13.43 | 185 |
|  | Cluster 2 | 14.07 | 4.27 | 15.10 | 60 |
|  | Cluster 3 | 12.82 | 7.80 | 20.06 | 100 |
|  | Cluster 4 | 11.31 | 10.97 | 30.64 | 124 |
|  | Cluster 5 | 10.46 | 5.64 | 26.14 | 59 |
|  | Cluster 6 | 8.60 | 9.54 | 26.06 | 82 |
|  | Cluster 7 | 7.61 | 7.89 | 14.29 | 60 |
|  | Cluster 8 | 6.67 | 12.00 | 31.95 | 80 |
|  | Cluster 9 | 4.55 | 16.72 | 34.26 | 76 |
|  | Cluster 10 | 3.86 | 19.19 | 36.90 | 74 |

Cluster density = (Number of elements) / (Average RMSD)

**Figure S3.**

Docking model structures of the RPA70N – pmRPA32 peptide complex from the major cluster in stereo view. (A) Docking model structure with RPA70N H80 – pmRPA32 Y9 as the contact pair. (B) Docking model structure with RPA70N H80 – pmRPA32 Y14 as the contact pair. The pmRPA32 peptide is displayed in green. The RPA70N H80 – pmRPA32 Y9 (A) and RPA70N H80 – pmRPA32 Y14 (B) contact pairs are colored in red. The residues within 4.5 Å of the pmRPA32 peptide are shown in blue.


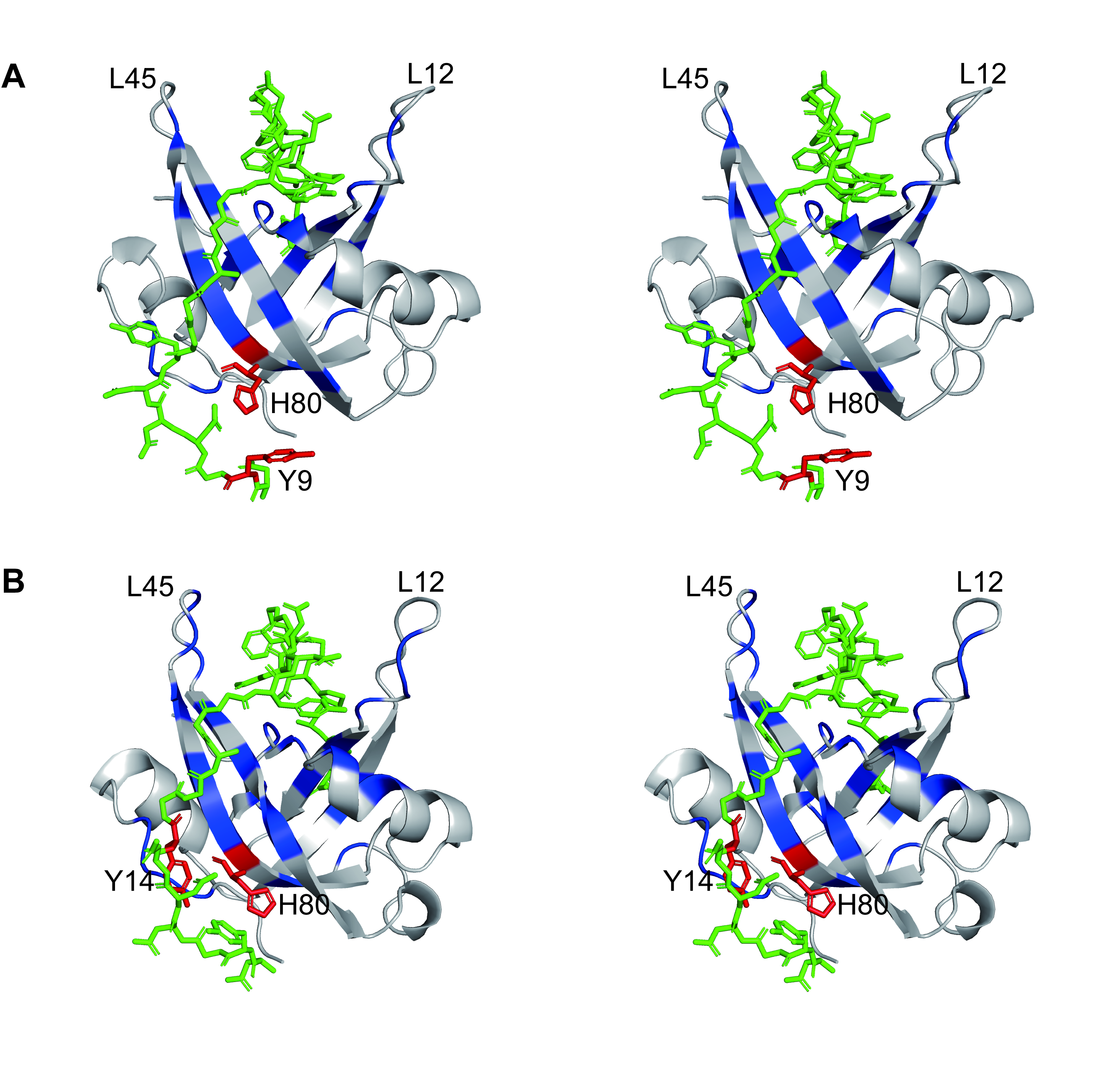


**Figure S4.**

Competitive fluorescence polarization anisotropy of BLM peptide, RPA70N, and pmRPA32 peptide. (A) Increasing concentrations of the pmRPA32 peptide were added to the FITC-labeled BLM_153-165_ and RPA70N complex. (B) Increasing concentrations of the pmRPA32 peptide were added to the FITC-labeled BLM_290-301_ and RPA70N complex.


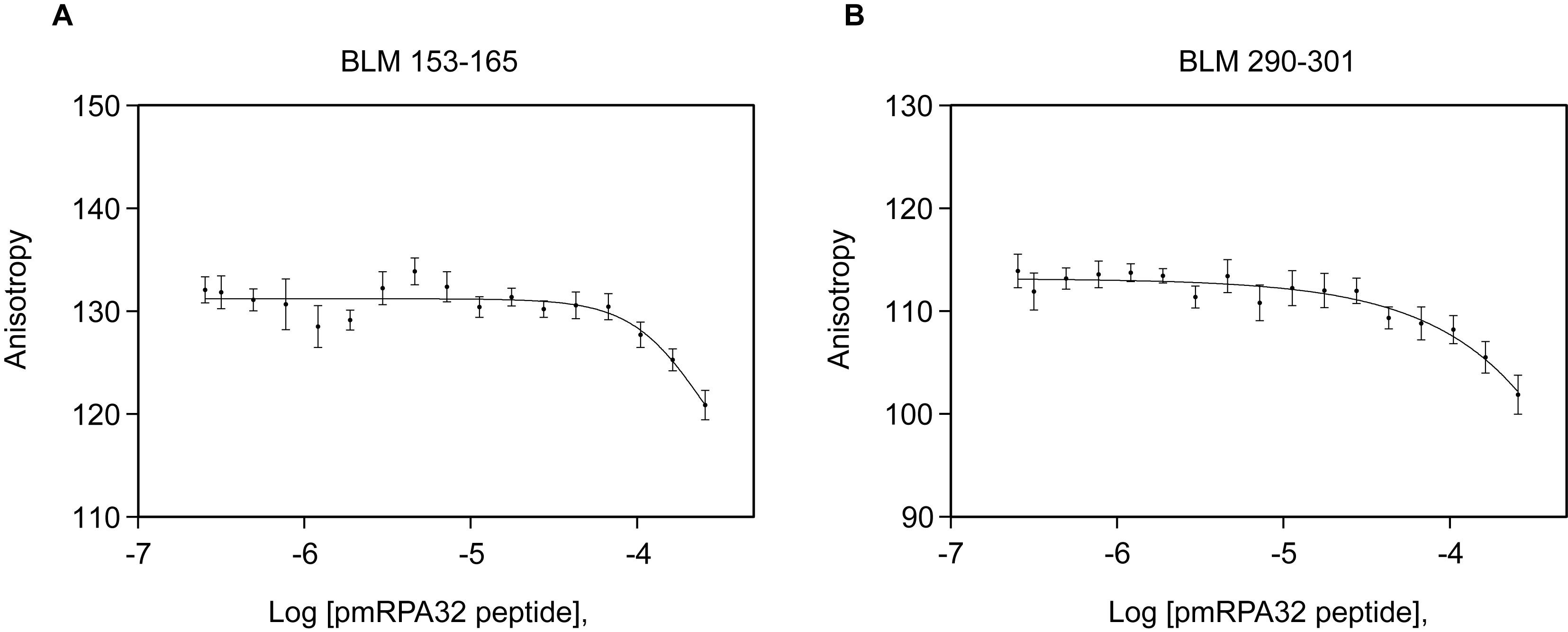

Supplement: Supplementary file 1 [file mmc1.docx]
